# Cardiovascular Effects of Splenomegaly and Splenectomy in Beta-Thalassemia Major

**DOI:** 10.7759/cureus.74186

**Published:** 2024-11-21

**Authors:** Sumira Abbas, Haris Ali Khan, Wajeeha Rehman, Mian Mufarih Shah, Muhammad Mustafa, Muhammad Abbas

**Affiliations:** 1 Pathology, Medical Teaching Institute-Hayatabad Medical Complex Peshawar, Peshawar, PAK; 2 Hematology and Pathology, Peshawar Medical College, Peshawar, PAK; 3 General Surgery, Aberdeen Royal Infirmary - NHS Grampian, Grampian, GBR; 4 Hematology and Pathology, Khyber Girls Medical College, Peshawar, PAK; 5 Medicine, Medical Teaching Institute-Hayatabad Medical Complex Peshawar, Peshawar, PAK; 6 Cardiology, Kuwait Teaching Hospital, Peshawar, PAK; 7 Cardiology, Peshawar Medical College, Peshawar, PAK; 8 Medicine, Peshawar Medical College, Peshawar, PAK; 9 Medicine, Prime Teaching Hospital, Peshawar, PAK

**Keywords:** beta-thalassemia major, blood pressure, cardiovascular function, ejection fraction, splenectomy, splenomegaly, thromboembolic events

## Abstract

Background

Beta-thalassemia major is a genetic blood disease complicated by splenomegaly, and splenectomy is a standard therapy for this medical condition. Although splenectomy results not only in the improvement of the hematological status, the long-term consequences to the cardiovascular system are still questionable.

Objective

The aim of the study was to assess and compare the cardiovascular impact of splenomegaly and splenectomy in patients with beta-thalassemia major.

Methodology

This is a cross-sectional survey conducted at Hayatabad Medical Complex, Peshawar, from January 2024 to June 2024. A total of 88 beta-thalassemia major patients were divided into two groups: 44 patients with splenomegaly were recruited and compared with 44 post-splenectomy patients. Outcomes that were assessed in this study from the medical records were cardiac function test, ejection fraction, left ventricular hypertrophy, thromboembolic incidences, pulmonary embolism, deep vein thrombosis, blood pressure level, and the biochemical markers, B-type natriuretic peptide (BNP) and troponin. The data were analyzed using the Statistical Package for the Social Sciences (SPSS) version 26 (IBM Corp., Armonk, USA).

Results

Patients with splenectomy exhibited slightly lower hemoglobin levels and higher transfusion requirements but had better ejection fractions compared to those with splenomegaly. In contrast, the splenomegaly group demonstrated worse cardiovascular outcomes, including higher rates of left ventricular hypertrophy, pulmonary hypertension, arrhythmias, thromboembolic events, and elevated BNP levels, indicating increased cardiovascular morbidity and hemodynamic strain. Logistic regression analysis further suggested that splenectomy reduces the risk of cardiomyopathy, particularly in younger patients, while the splenomegaly group experiences a greater burden of acute cardiovascular complications.

Conclusion

Splenectomy in beta-thalassemia major patients offers improved cardiac function and hemoglobin levels while reducing transfusion needs, but it does not eliminate the risks of long-term cardiovascular and thromboembolic complications, such as deep vein thrombosis and pulmonary embolism. On the other hand, patients with splenomegaly face more severe and immediate cardiovascular risks, including hypertension, arrhythmias, and thromboembolic events. These findings underscore the importance of tailored management strategies to optimize outcomes for both groups.

## Introduction

Beta-thalassemia major represents one of the severe forms of inherited hemolytic anemia due to reduced or absent synthesis of beta-globin chains in hemoglobin [1]. Consequently, patients with beta-thalassemia major have compromised erythropoiesis and need periodic blood transfusions to maintain normal hemoglobin levels or even elevated levels, but this approach causes an increased risk of iron toxicity and subsequent organ dysfunction [2, 3]. This disease tends to affect the spleen and leads to splenomegaly, which is a common occurrence in patients diagnosed with beta-thalassemia major [4-6].

Splenomegaly is caused by increased rates of red blood cell destruction and extramedullary hematopoiesis [7, 8]. This continuous sequestration harms the anemic patient by further exacerbating the anemia, increasing the need for transfusions, and enhancing hemolysis. To address this, splenectomy, which is the process of removing the spleen, may be conducted. Splenectomy is common when hypersplenism causes splenomegaly that results in excessive filtration of blood components, thus provoking severe anemia, leukopenia, or thrombocytopenia [9-11]. While splenectomy has been shown to significantly reduce transfusion requirement and improve haematologic profile, these come with some risks, especially in the cardiovascular system [12, 13].

Cardiac diseases in beta-thalassemia major are prevalent and heterogeneous, resulting from anemia, iron overload, and splenic factors [14]. The heart is chronically exposed to dumped iron leading to cardiomyopathy, arrhythmia, and congestive heart failure. Moreover, splenectomy patients may be prone to thrombotic disorders, which will cause diseases such as pulmonary hypertension and venous thromboembolism (VTE), which make cardiovascular health worse [15, 16].

It is imperative to consider the cardiovascular impacts of splenomegaly and splenectomy in patients with beta-thalassemia major as these factors may affect later prognosis. Splenomegaly itself plays an important role in hyperdynamic circulation, which works its way to enhance cardiac output, leading to left ventricle hypertrophy and heart failure if the problem persists [17]. Splenectomy in beta-thalassemia major patients improves blood transfusion rates and hemoglobin levels, as shown by studies including Osataphan et al. [13], who found that splenectomy reduces blood transfusion requirements in patients with TDT, and Ikram et al. [18]. However, while hematological improvements are evident, splenectomy is associated with increased iron burden and higher serum ferritin levels, as noted in research by Zhou et al. [19]. Still, splenectomy exhibits the risk of postoperative infections, thrombosis, and late adverse cardiovascular effects resulting from impaired splenic filtration of abnormal cells and prothrombotic particulate matter.

The present study is conducted to assess the impact of splenomegaly on the cardiovascular systems and after splenectomy in beta-thalassemia major patients. The study will primarily compare cardiac function and hemodynamic status, as well as the rate of thromboembolic events in splenomegaly and post-splenectomy patients. This research aims to establish a correlation between conservative management or splenectomy in beta-thalassemia major patients and its effect on cardiovascular status to help manage the condition and improve patients’ prognosis.

Understanding the links between splenomegaly, splenectomy, and cardiovascular events is critical to controlling the severity of beta-thalassemia major and reducing mortality in patients. We also assume that this study will help increase the current scanty information concerning cardiovascular complications linked with this hematological disorder. Therefore, the goal of the present work is to assess the cardiovascular consequences of splenomegaly and splenectomy, cardiac dysfunction, hemodynamic alterations in patients with beta-thalassemia major, and frequencies of thromboembolism as well as to give ideas about their management.

## Materials and methods

In this research work, participants of this study constitute patients with splenomegaly and those who have undergone splenectomy because of beta-thalassemia major diagnosis. The study aimed to assess cardiac function, changes in hemodynamics, and thromboembolic risks in splenectomised and nonsplenectomised patients. The study was conducted at the Medicine OPD, Hayatabad Medical Complex, Peshawar. The study was conducted in six months, starting from January 2024 to June 2024.

A total of 88 patients with beta-thalassemia major were included in the study. The main approach to sample selection in this study was consecutive sampling. This approach made certain that all the records of beta-thalassemia major patients who were eligible and available at the Medicine OPD of the Hayatabad Medical Complex during the research period were involved in the study. However, data from these patients were obtained from a review of their case files obtained during their hospitalization. A non-probability sampling technique was carried out, which included a consecutive sampling technique where the researchers were able to enroll all the cases that met specific inclusion criteria within a defined time period.

The beta-thalassemia major patients included were those between 10 and 25 years, with splenomegaly or history of splenectomy, and on regular blood transfusion for at least one year. Patients with congenital heart diseases or other chronic disorders not associated with thalassemia, those who had a prior history of thromboembolic episodes not due to thalassemia or splenectomy, patients who underwent splenectomy not for splenomegaly resulting from beta-thalassemia major comprise the exclusion criteria of the evaluation. The retrieval of the data was done in such a way that only those patients whose records were complete with the information required were selected.

Data were collected from medical records, patient interviews, and clinical assessments at the hospital. All patients with beta-thalassemia major were identified from the hospital’s records. Information on the patient’s age, gender, splenomegaly history, whether the patient has undergone splenectomy, and their transfusion history was obtained from hospital records. Information on clinical parameters, which includes packed cell volume, total blood transfused, and period of blood transfusion, was also recorded.

Cardiovascular parameters, including echocardiogram, ECG, and blood pressure measurement, were also recorded to check for any sign of hypertrophy, cardiomyopathy and arrhythmia, hypertensive or pulmonary hypertensive signs, and conduction disorder, respectively. Pulmonary hypertension was defined by the presence of pulmonary arterial systolic pressure greater than or equal to 30 mmHg by transthoracic echocardiography. The diagnosis of Pulmonary Hypertension was made using the tricuspid regurgitation velocity using a systolic pulmonary arterial pressure (sPAP) of ≥ 35 mmHg. Intrarater reliability of echocardiographic assessment was obtained by using experienced cardiologists to measure the variable and thus reduce variability across the subjects.

Blood samples were also collected to determine blood cardiac stress and injury enzymes which are troponins and B-type natriuretic peptides. For deep vein thrombosis (DVT), pulmonary embolism, or any other thromboembolic disorders, clinical history and Doppler ultrasonography of the involved area were done**. **In patients in the splenomegaly group, the size of the spleen was measured by ultrasound abdomen.** **By the patients who had splenectomy, the duration after surgery and the presence and type of complications after the operation, for example, thrombosis, were taken.

All the data were recorded and analyzed on the Statistical Package for Social Sciences (SPSS) 26 (IBM Corp., Armonk, USA). Basic statistics involving distribution frequency, percentage of categorical variables, and mean ± standard deviation of the numeric variables were used. Patients were divided into two groups: those with splenomegaly and those who had splenectomy done on them. Multivariable regression analysis was conducted to investigate splenectomy's effects on cardiovascular outcomes and control the possibility of confounding factors. First, the models accounted for age, gender, and transferral behaviors to allow the effects of splenectomy to be seen independently of these factors. These included binary logistic regression for binary dependent variables, including the presence or absence of cardiomyopathy and arrhythmias linear regression, on the other hand, was used for continuous variables, including the score of the echocardiogram of the cardiac function and then checking for interaction with age and gender to see if the effect of splenectomy was different in the two variables. Furthermore, age, gender, splenomegaly history, and transfusion frequency were kept as covariates in each model to establish that these are actual effects of splenectomy. As such, the adjusted models afford a strong statistical foundation to quantify the direct associations between splenectomy and cardiovascular health outcomes.

The ethical approval for this research was transferred from the Institutional Research and Ethical Board of Hayatabad Medical Complex, Khyber Pakhtunkhwa, Pakistan. The consent and anonymity of all participants were maintained throughout the study. To ensure that patients’ identities were not compromised, all data collected were anonymized, and patients were informed of their right to volunteer to participate in the study and could withdraw without repercussions on their medical treatment.

## Results

In the present cross-sectional study, the population included two groups of patients: one group consisted of patients who had undergone splenectomy, and the other consisted of patients who had splenomegaly. The age and gender differences between the two groups were almost similar. However, a comparison of the average hemoglobin levels and the frequency of blood transfusions between the splenectomy group and the splenomegaly group showed that patients in the splenectomy group had slightly lower average hemoglobin levels and higher demands for blood transfusions after splenectomy.

The splenomegaly group had received blood transfusions more frequently, indicating a longer duration of the disease before surgery and greater transfusion needs prior to splenectomy. The splenomegaly group also presented with a significantly larger spleen size, with an average of 16.3 cm. Patients in the splenectomy group had, on average, been surviving for 4.6 years post-operation, indicating how much they had adapted to life after the surgery, as shown in Table 1.

**Table 1:** Demographic and Clinical Characteristics of the Study Population

Characteristic	Splenectomy Group (n=44)	Splenomegaly Group (n=44)
Age (years)	20.2 ± 6.3	21.5 ± 5.8
Gender (Male/Female)	24/20	25/19
Hemoglobin (g/dL)	8.6 ± 1.4	9.3 ± 1.6
Blood Transfusions (per year)	18.6 ± 4.2	15.3 ± 3.7
Duration of Blood Transfusions (years)	12.9 ± 4.8	14.4 ± 5.2
Spleen Size (cm)	N/A	16.3 ± 3.6
Time Since Splenectomy (years)	4.6 ± 2.3	N/A

When comparing the cardiovascular assessment of splenectomy to splenomegaly, certain dissimilarities were observed. The splenectomy group yields a slightly higher mean ejection fraction, which indicated comparatively superior cardiac performance than the splenomegaly group. Whereas left ventricular hypertrophy, as well as pulmonary hypertension, is more evident in the splenomegaly group, suggesting that cardiac load is higher in this subgroup of patients. Hypertension and frequent arrhythmias are also more likely to be observed in the splenomegaly group, and thromboembolic events had a higher likelihood of occurring with increased splenomegaly, suggesting a higher potential for clotting complications. Also, there was a higher concentration of BNP (B-type natriuretic peptide), which reflects heart failure or cardiac stress, in the splenomegaly group in contrast to the splenectomy group. Based on these studies, it can be postulated that cardiovascular complications are worse in patients with splenomegaly than in splenectomized patients(Table 2).

**Table 2:** Cardiovascular Assessment Results BNP: B-type natriuretic peptide

Cardiovascular Parameter	Splenectomy Group (n=44)	Splenomegaly Group (n=44)
Ejection Fraction (%)	55.4 ± 7.2	52.2 ± 6.9
Left Ventricular Hypertrophy	11 (25%)	15 (34.0%)
Pulmonary Hypertension	9 (20.4%)	13 (29.5%)
Arrhythmias	5 (11.3%)	7 (15.9%)
Thromboembolic Events	4 (9.0%)	10 (22.7%)
BNP Levels (pg/mL)	85.1 ± 21.5	111.6 ± 27.8

The biomarker and event data show different temporal patterns between the splenectomy and splenomegaly groups. Serum troponin I levels, an index of cardiac damage, were slightly elevated in the splenomegaly group, but all were within the normal range in both groups. The mean blood pressure in the splenomegaly group was also significantly higher than in the splenectomy group for both systolic as well as diastolic pressure. Median systolic blood pressure and heart rate were higher in the splenomegaly group, suggesting excess cardiac workload and demand.

More complications related to clotting are evident in the splenomegaly group, implying more DVT and PE. On the other hand, the splenectomy group had a comparatively fewer occurrence of these events. These observations lead to the conclusion that patients with splenomegaly are at higher cardiovascular and thromboembolic risk as compared to patients following splenectomy (Table 3).

**Table 3:** Cardiac Biomarkers, Hemodynamic Parameters, and Thromboembolic Event Distribution

Biomarker/Parameter/Events	Splenectomy Group (n=44)	Splenomegaly Group (n=44)
Troponin I (ng/mL)	0.03 ± 0.02	0.04 ± 0.01
Systolic BP (mmHg)	110.7 ± 12.2	119.4 ± 13.6
Diastolic BP (mmHg)	74.3 ± 9.5	78.8 ± 10.3
Heart Rate (beats/min)	84.4 ± 10.5	88.3 ± 12.4
Deep Vein Thrombosis (DVT)	2 (4.54%)	5 (11.3%)
Pulmonary Embolism (PE)	2 (4.54%)	5 (11.3%)
Other Thromboembolic Events	0 (0%)	1 (2.2%)

The logistic regression model aimed to predict the presence of cardiomyopathy based on several factors: splenectomy, age, gender, the number of transfusions, and the interactions between splenectomy and both age and gender. According to their findings, splenectomy had an effect of a decreased risk of cardiomyopathy (p = 0.028). Further analysis showed that there was a significant interaction between splenectomy and age (p=0.036), meaning that the risk of getting cardiomyopathy after splenectomy depends on age. There was a slight decrease in the preventative role of splenectomy as age advanced. Therefore, gender and transfusion frequency could not be used to predict cardiomyopathy, and neither could splenectomy and gender have an interactive effect (Table 4).

**Table 4:** Logistic Regression Analysis for Predicting Cardiomyopathy

Variable	Coefficient (β)	Standard Error	Odds Ratio (OR)	p-value
Splenectomy	-0.733	0.333	0.482	0.028
Age	0.025	0.018	1.025	0.146
Gender	-0.206	0.292	0.816	0.477
Number of Transfusions	0.090	0.123	1.094	0.457
Splenectomy × Age Interaction	0.016	0.008	1.016	0.036
Splenectomy × Gender Interaction	0.113	0.199	1.120	0.575

**Figure 1:**
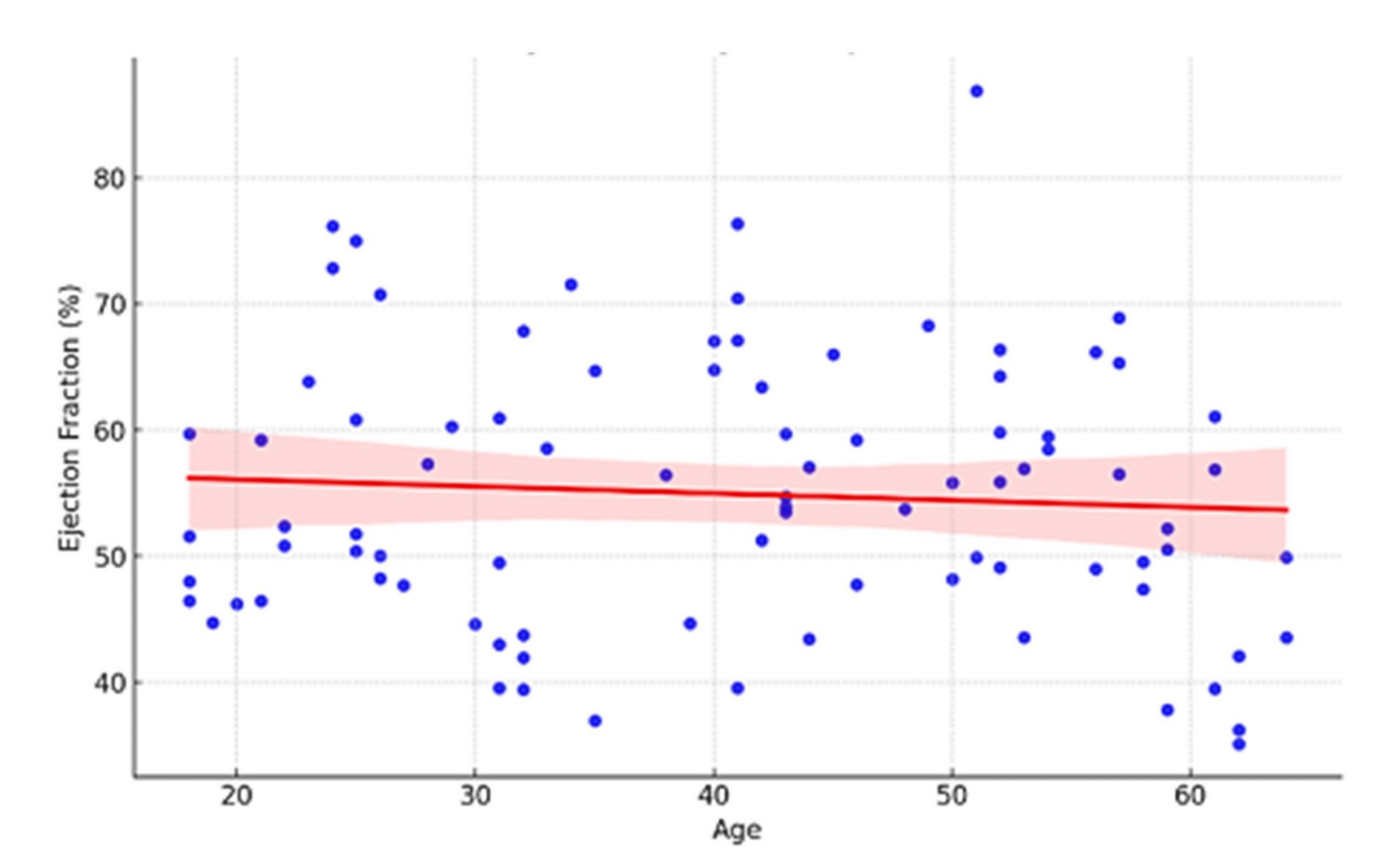
The Relationship between Age and Ejection Fraction by Linear Regression Model

The linear regression model aimed at evaluating the effects of the same factors on ejection fraction, a continuous cardiovascular measure. Thus, no factors were identified that were associated with the changes in ejection fraction, such as splenectomy, age, gender, frequency of transfusions, or their interaction. This implies that these factors do not exert much, if any, direct effect on ejection fraction. Nevertheless, there was a slight trend towards the significance of the interaction between splenectomy and gender (F = 3.47, p = 0.114), which suggested that the effect of splenectomy on ejection fraction may be different in the two genders. Although age was weakly negatively associated with ejection fraction, no clear curvilinear trend was evident, indicating that age may not necessarily affect ejection fraction in this population (Figure 1).

## Discussion

The current study aimed to evaluate the impact of splenomegaly and splenectomy on the cardiovascular system and cardiac function, thromboembolic episodes, and hemodynamics in patients with beta-thalassemia major. As illustrated by the findings in this study, patients with beta-thalassemia major who undergo splenectomy experience fewer cardiovascular complications compared to those with splenomegaly. This is supported by observations of a higher ejection fraction, fewer thromboembolic events, and better blood pressure control in splenectomized patients. These results suggest that splenectomy may play a protective role in mitigating cardiovascular risks, possibly by reducing chronic inflammation or iron overload associated with splenomegaly.

The patients in the splenectomy group had lower average mean hemoglobin levels and a higher rate of blood transfusion than those in the splenomegaly group, consistent with the literature regarding the function of the spleen in filtering out abnormally shaped red blood cells and preventing hemolysis in the splenectomy population (Zafeiropoulos et al., 2024) [20]. Similarly, Wood JC et al. (2023) reported that splenectomy for thalassemia brings about cardiovascular remodeling, which leads to left ventricular hypertrophy, as revealed by increased prevalence amongst the subjects under study [21]. Furthermore, it is stated that even though splenectomy decreases hemoglobin concentration due to the removal of the major site of red blood cell destruction, there is an increase in transfusion dependency over time. This phenomenon can be attributed to a reduction of the spleen in erythrocyte sequestration and increased erythrocyte destruction in the peripheral circulation. The increased transfusion rate in our splenectomy group is in harmony with these findings, which suggests that the spleen plays a multifaceted role in maintaining hemoglobin balance in beta-thalassemia major.

Conversely, Meloni A et al. (2024) did not find any statistically significant difference in the rates of ejection fraction between splenectomized and non-splenectomized thalassemia major patients, while, in the present study, low ejection fraction has been found in splenectomy group [22]. These differences may arise from variations in participant demographics, such as age at splenectomy, and methodological differences, including the timing of ejection fraction assessments and imaging techniques used. Additionally, differences in treatment protocols, like iron chelation and transfusion practices, could influence cardiac outcomes. These factors suggest the need for careful consideration of patient management and study design in interpreting results across different studies. Contrary to our study, Ho G et al. (2020) found that splenectomy increases the identification of thromboembolic complications; such complications are less common in the present study [23]. The differences may be attributable to variations in the management and the follow-up, as well as the use of prophylactic anticoagulation, which has not been addressed in the present study.

In contrast to our findings, which showed left ventricular hypertrophy and pulmonary hypertension being more prominent in the splenomegaly group, Caocci et al. (2023) reported a higher prevalence of pulmonary hypertension in splenectomized thalassemia patients due to raised vascular resistance and hemodynamic changes from platelet activation [24]. In contrast, Bonaguro AM et al. (2023) did not find any significant increase in pulmonary hypertension after splenectomy [25]. This difference could be explained by disparities in patient care concerning chelation therapy, which has been shown to affect pulmonary status. For instance, a change in the life course of these patients may be achievable through the use of chelation therapy to lessen the iron load and possible vascular problems that lead to pulmonary hypertension.

In contrast, a study by Tahir F et al. (2020) observed an elevated risk of thromboembolic events such as PE and DVT in splenectomy patients, while these events had an increased prevalence in the splenomegaly group of our study participants [26]. This is consistent with McPhetridge JB et al. (2022) who reported that changes in platelet turnover and endothelial functions cause coagulopathy following splenectomy [27]. Different study populations and brief follow-up periods without well-stated standardization of anticoagulation could be the cause of discrepancies in reported outcomes in the studies. Such examination is imperative to develop a better insight into the factors that predispose the patients to thromboembolism risks after splenectomy and the medical approaches that can prevent the occurrence of the aforementioned complications.

Our findings show increased cardiac dysfunction in splenectomy patients compared to Daar S et al. (2023), where more cardiac complications are recorded among splenomegaly patients [28]. The observed discrepancy could be attributed to spleen size and blood supply, features not initially considered in this study. Splenic size can also affect the hemodynamic indicators; the greater the splenomegaly, the greater the effect on blood flow and vascular resistance and, consequently, cardiac performance. Furthermore, the impact of these effects may be dose-dependent or patient-specific, and the conditions of patients in our study population were not always severe. These findings are in agreement with the study by Saadatifar H et al. (2021), demonstrating high cardiac stress markers among splenectomized thalassemia patients, indicating subclinical heart failure [29]. As highlighted by these findings, echocardiographic examination should always be included in splenectomy outcomes assessment. Stanca I et al. (2021) also proposed BNP as a prognostic indicator [30].

Altogether, the results of this research suggest that cardiovascular complications, including cardiac dysfunction and thromboembolic processes, are less pronounced in splenectomized beta-thalassemia major patients compared to those with splenomegaly, highlighting the potential mitigating effects of splenectomy on cardiovascular risks.** **However, the differences in the results of the study may be explained by different characteristics of patients and surgical and postoperative treatment methods. These findings highlight the need for individualized medical management, more so in patients who have undergone splenectomy where there is an accumulation of cardiovascular risk factors. Further improvement for patient management aims for future studies, which should regard possible long-term cardiovascular effects of splenectomy taking recourse to epidemiological cohort prospective design.

Furthermore, comparative research concerning various post-splenectomy regimens, which include distinct anticoagulants and chelating agents, may help with the determination of the most effective therapy to offer beta-thalassemia major patients. The act of performing such specific research will be important in establishing set standards and efficient post-surgical care protocols. Clinical data on cardiac functioning and thromboembolic risks prove to be an effective basis for the conclusions made regarding the effects of splenectomy on health. Furthermore, the comparisons made between splenectomized and non-splenectomized groups enable us to understand the post-operative effects of such surgery in detail. However, some limitations should be noted.

However, the retrospective design of the present study weakens the evidence of causality between splenectomy and the health outcomes identified in the study. Procedures performed in the treatment of patients included in the analysis may have varied treatment regimens regarding chelation therapy and anticoagulation treatment, which can affect the results and lessen inter-group comparability. Lastly, the duration of observed follow-up may have been too short to detect long-term endpoints, including the risk of late-onset complications. Finally, since the present studies employed different measures and instruments across sites, this may have contributed to measurement bias that could compromise the generalizability of the results.

## Conclusions

This study demonstrates that splenectomy in beta-thalassemia major patients is associated with better cardiac function, as evidenced by improved ejection fractions and reduced risk of left ventricular hypertrophy compared to patients with splenomegaly. However, splenectomy also increases the risk of thromboembolic complications such as deep vein thrombosis and pulmonary embolism, particularly in older patients. Conversely, patients with splenomegaly exhibit worse overall cardiovascular outcomes, including higher rates of hypertension, arrhythmias, thromboembolic events, and elevated biomarkers, indicating a greater cardiac burden. These findings suggest that while splenectomy offers some cardiovascular benefits, it also comes with specific risks that must be carefully managed. Future studies should further explore these differences to optimize management strategies for beta-thalassemia major patients.

## References

[1] Shafique F, Ali S, Almansouri T (2021). Thalassemia, a human blood disorder. Braz J Biol.

[2] Pinto VM, Forni GL (2020). Management of iron overload in beta-thalassemia patients: clinical practice update based on case series. Int J Mol Sci.

[3] Sharif Y, Irshad S, Muazzam A (2021). Assessment of patients with β-thalassemia major, undergoing tertiary care at a regional thalassemia center in Pakistan. Pak J Zool.

[4] Cenariu D, Iluta S, Zimta AA (2021). Extramedullary hematopoiesis of the liver and spleen. J Clin Med.

[5] Peretz S, Livshits L, Pretorius E (2022). The protective effect of the spleen in sickle cell patients. A comparative study between patients with asplenia/hyposplenism and hypersplenism. Front Physiol.

[6] Azeez FS (2022). Effect of Splenectomy in thalassemia major patients on some blood parameters, hormones & blood transfusion frequency. Sumer Univ J Pure Sci.

[7] Chauhan W, Shoaib S, Fatma R, Zaka-Ur-Rab Z, Afzal M (2022). Beta-thalassemia and the advent of new interventions beyond transfusion and iron chelation. Br J Clin Pharmacol.

[8] Ortega-Paz L, Capodanno D, Montalescot G, Angiolillo DJ (2021). Coronavirus disease 2019-associated thrombosis and coagulopathy: review of the pathophysiological characteristics and implications for antithrombotic management. J Am Heart Assoc.

[9] Godeau B (2023). Is splenectomy a good strategy for refractory immune thrombocytopenia in adults?. Br J Haematol.

[10] Capecchi M, Ciavarella A, Artoni A, Abbattista M, Martinelli I (2021). Thrombotic complications in patients with immune-mediated hemolysis. J Clin Med.

[11] Du L, Deng H, Wu X, Liu F, Yin T, Zheng J (2024). Relationship between spleen pathologic changes and spleen stiffness in portal hypertension rat model. Ultrasound Med Biol.

[12] Akca T, Ozdemir GN, Aycicek A, Ozkaya G (2023). Long-term results of splenectomy in transfusion-dependent thalassemia. J Pediatr Hematol Oncol.

[13] Osataphan N, Dumnil S, Tantiworawit A (2023). The long-term efficacy in blood transfusions, hematologic parameter changes, and complications after splenectomy in patients with transfusion-dependent thalassemia. Transfus Apher Sci.

[14] Arumugaswamy PR, Singh D, Rathore YS (2023). Chapter 3: Splenectomy: clinical applications and outcomes. Advances in Health and Disease.

[15] Mancardi D, Mezzanotte M, Arrigo E, Barinotti A, Roetto A (2021). Iron overload, oxidative stress, and ferroptosis in the failing heart and liver. Antioxidants (Basel).

[16] Kontoghiorghes GJ (2023). Iron load toxicity in medicine: from molecular and cellular aspects to clinical implications. Int J Mol Sci.

[17] Sousa L, Oliveira MM, Pessôa MT, Barbosa LA (2020). Iron overload: effects on cellular biochemistry. Clin Chim Acta.

[18] Ikram N, Anwar T, Gul-e-Najaf Gul-e-Najaf, Ahmed B, Subhani FA. (2022). Splenectomy in Patients with Beta Thalassaemia Major. J haematol stem cell res.

[19] Zhou YL, Zhang XH, Liu TN, Wang L, Yin XL (2014). Splenectomy improves anaemia but does not reduce iron burden in patients with haemoglobin H Constant Spring disease. Blood Transfus.

[20] Zafeiropoulos S, Ahmed U, Bekiaridou A (2024). Ultrasound neuromodulation of an anti-inflammatory pathway at the spleen improves experimental pulmonary hypertension. Circ Res.

[21] Wood JC (2023). Cardiac complications in thalassemia throughout the lifespan: victories and challenges. Ann N Y Acad Sci.

[22] Meloni A, Pistoia L, Ricchi P (2024). Multiparametric cardiac magnetic resonance in patients with thalassemia intermedia: new insights from the E-MIOT network. Radiol Med.

[23] Ho G, Brunson A, Keegan THM, Wun T (2020). Splenectomy and the incidence of venous thromboembolism and sepsis in patients with autoimmune hemolytic anemia. Blood Cells Mol Dis.

[24] Caocci G, Mulas O, Barella S (2023). Long-term health-related quality of life and clinical outcomes in patients with β-thalassemia after splenectomy. J Clin Med.

[25] Bonaguro AMG, Walsh C, Leghari MO (2023). A case of post-splenectomy pulmonary hypertension. Chest.

[26] Tahir F, Ahmed J, Malik F (2020). Post-splenectomy sepsis: a review of the literature. Cureus.

[27] McPhetridge JB, Lynch AM, Webster CR, McCobb E, de Laforcade AM, O'Toole TE (2022). Pre-operative hemostatic status in dogs undergoing splenectomy for splenic masses. Front Vet Sci.

[28] Daar S, Taher A (2023). Chapter 12: Splenomegaly and splenectomy. 2021 Guidelines: For the Management of Transfusion Dependent Thalassaemia (TDT) [Internet], 4th edition.

[29] Saadatifar H, Niayeshfar A, Mard-Soltani M, Bahrampour E, Khalili S, Alinezhad Dezfuli D, Pouriamehr S (2022). The correlation of cardiac biomarkers and myocardial iron overload based on T2* MRI in major beta-thalassemia. Int J Cardiovasc Imaging.

[30] Stanca I, Rus M, Albu A, Fica S (2020). Predictive factors of heart failure in patients with beta-thalassemia major. Technium Soc Sci J.

